# Dataset of research misconduct knowledge and associated factors among nurses in China: A national cross-sectional survey

**DOI:** 10.1016/j.dib.2022.108471

**Published:** 2022-07-16

**Authors:** Shuyu Han, Ke Li, Zhiwen Wang

**Affiliations:** a School of Nursing, Peking University, Beijing 100191, China; b Department of Emergency, Peking University First Hospital, Beijing 100034, China; c Peking University Health Science Centre for Evidence-Based Nursing: A Joanna Briggs Institute Affiliated Group, Beijing 100191, China

**Keywords:** Nurse, Research misconduct, National survey, Knowledge, Continuing training

## Abstract

Engagement in research misconduct by nurses may results in professional misconduct in the clinical setting, thereby jeopardizing the quality of patient care. We still know little about the research misconduct situation among nurses. Previous attempts also hardly reflected participants’ actual knowledge level of research misconduct. This data article presents a novel dataset of a cross-sectional study investigating the research misconduct knowledge level and associated factors among nurses in China. Between March 2018 and March 2021, a national survey was conducted at 200 tertiary hospitals in 25 provinces. A multistage sampling (province, hospital, and participants) was applied and 4,112 nurses were recruited in this study. Participants completed questionnaires online through smartphones scanning a Quick Response (QR) code. The survey consisted of demographic characteristics, research activities, scientific misconduct knowledge, perceived reasons for research misconduct and perceived consequences for research misconduct. Data from 3,640 nurses were reserved in the dataset after data cleaning. This dataset may provide comprehensive information on research misconduct knowledge and associated factors, and important evidence for designing research integrity continuing training for nurses.

## Specifications Table

**Table:** 

Subject	Nursing and Health Professions
Specific subject area	Research misconduct knowledge level among nurses
Type of data	Table
How the data were acquired	Participants completed questionnaires online through smartphones scanning a Quick Response (QR) code that included the informed consent on the home page and all the items. The questionnaire can only be started when an “Agreement” is checked in the informed consent form. The questionnaire and code book are available in Appendix I and Appendix II.
Data format	RawAnalyzed
Description of data collection	Sampling followed a tiered process, which included three levels: province, hospital, and participants. Of the 31 provinces in China, we involved all four province-level municipalities (Beijing, Shanghai, Tianjin, and Chongqing) and chose three provinces in each Chinese geographical division (Northeast Region, North Region, East Region, South Region, Central Region, Northwest Region, and Southwest Region). The sampling frame did not include Hong Kong, Macao, and Taiwan. We then conducted purpose sampling in these 25 provinces. Eight hospitals were sampled in each province, including one teaching hospital, one province-level comprehensive hospital, three specialized hospitals (such as Obstetrics and Gynecology hospital, Pediatric hospital, Dental hospital, Oncology hospital etc.), one traditional Chinese medicine hospital, and two city-level comprehensive hospitals. A total of 200 hospitals were included in our study. Finally, under the coordination of provincial and municipal hospital management centers, a convenience sampling method was used to recruit participants in each hospital. The data collection date was between March 2018 and March 2021.
Data source location	• Institution: 200 tertiary hospitals in 25 provinces • Country: China
Data accessibility	The raw data is available in the Mendeley data repository at https://data.mendeley.com/datasets/chx6dr7bpw/1[1].

## Value of the Data

• This data help acquire knowledge of research misconduct knowledge level among nurse in China.
• This data enables researchers to further investigate the factors that influence research misconduct knowledge.
• This data can provide evidence for designing research integrity continuing training for nurses.
• This data can assist hospital managers and policy makers in taking effective measures to improve research misconduct knowledge of nurses.

## 1 Data Description

Many previous studies have reported the prevalence of research misconduct among nursing students [2], [3]. However, we still know little about the research misconduct situation among nurses. Previous attempts also hardly reflected participants’ actual knowledge level of research misconduct. Some studies just asked participants if they knew about relevant research misconduct knowledge [4], [5]. Therefore, we designed a questionnaire according to two official materials [6], [7] to measure the knowledge level of research misconduct among nurses.

Between March 2018 and March 2021, a total of 4,112 nurses participated in our study. All the data was collected through online questionnaires. We designed in the online questionnaire that incomplete questionnaires cannot be submitted, so there are no missing values in the database. According to our pilot study, we estimated that the questionnaire filling process usually required approximately 10-15 minutes to complete. Therefore, we excluded those questionnaires from filling out time records in the system that were less than 5 minutes long. We also excluded records that responded in straight lines. Finally, data from 3,640 participants were used for data analysis.

As shown in Table 1, the average age of the participants was 38.27 ± 7.85, and their average years of career was 16.51 ± 9.12. The majority of them were female (96.6%), married (84.0%), and had children (79.8%). More than 80% of the participants had a Bachelor's degree or above. Two-thirds (67.0%) of participants were in formal employment. Nearly 70% of the participants (69.3%) were entitled to be a nurse-in-charge. Most of the participants (91%) came from Grade A tertiary hospitals. Three quarters of them reported they were in a clinical position.

**Table 1:** Participant characteristics (*N* = 3640).

Characteristics	*N* (%), M ± SD (IQR)
Age	38.27 ± 7.85 (20 - 65)
Gender	
Male	123 (3.4)
Female	3517 (96.6)
Marital status	
Unmarried	584 (16.0)
Married	3056 (84.0)
Years of career	16.51 ± 9.12 (0 - 48)
Fertility status	
No	734 (20.2)
Yes	2906 (79.8)
Educational attainment	
College or less	636 (17.5)
Bachelor degree	2642 (72.6)
Master degree or above	362 (9.9)
Employment situation	
Formal nurses	2439 (67.0)
Informal nurses	1201 (33.0)
Title	
Nurse	211 (5.8)
Nurse practitioner	572 (15.7)
Nurse-in-charge	2523 (69.3)
Associate director of nursing	285 (7.8)
Director of nursing	49 (1.3)
Institution level	
Grade A tertiary hospital	3312 (91.0)
Grade B tertiary hospital	267 (7.3)
Grade C tertiary hospital	61 (1.7)
Department	
Clinical department	3040 (83.5)
Other	600 (16.5)
Position	
Clinical position	2759 (75.8)
Research position	74 (2.0)
Management position	760 (20.9)
Service position	290 (8.0)

The average score of research misconduct knowledge among the participants was 15.99 ± 5.79 (range 0 to 30). The descriptive analyses of research activities, perceived reasons for research misconduct, and perceived consequences for research misconduct are shown in Tables 2–Table 4, respectively. Of the 11 listed research activities, the top three with the highest percentages were “had patents”, “had participated in research projects as other roles”, and “was a PI for a research project” (Table 2). Approximately one-third (34.5%) of the participants reported that they had published academic papers as the first author or corresponding author. However, only 3.5% of the participants had published SCIE-indexed papers as the first author or corresponding author. The average research activity index of the participants was 3.04 ± 1.62.

**Table 2:** Participants' research activities (*N* = 3640).

Research activities	*N* (%), M ± SD (IQR)
I have published academic papers as the first author or corresponding author	1254 (34.5)
I have published SCIE-indexed papers as the first author or corresponding author	126 (3.5)
I have published books as an editor-in-chief	156 (4.3)
I have published books as an editorial board member	422 (11.6)
I am a primary investigator (PI) for a research project	1612 (44.3)
I have participated in research projects in other roles (non-PI)	1961 (53.9)
I have won research awards	1465 (40.2)
I have patents	2387 (65.6)
I have attended academic conferences and have given oral or poster presentations	1602 (44)
I am a reviewer for an academic journal	42 (1.2)
I am an editorial board member for an academic journal	45 (1.2)
Research activity index	3.04 ± 1.62 (1 - 11)

Note. SCIE-indexed: indexed by the Science Citation Index-Expand (SCIE) database.

**Table 3:** Perceived reasons for research misconduct (*N* = 3640).

Item	*N* (%)
Nurses deviate in personal value and lack of academic ethics	2868 (78.8)
Nurses lack research ability	2454 (67.4)
There is a lack of research integrity training	2200 (60.4)
Nurses do not understand the content of research integrity	2315 (63.6)
There is a lack of academic supervision	2644 (72.6)
There exist defects of academic quantitative evaluation	2531 (69.5)
Nurse are influenced by social environment	2171 (59.6)

**Table 4:** Perceived consequences for research misconduct (*N* = 3640).

	N(%)					
Item	No influence	A little influence	Moderate influence	Strong influence	Very strong influence	M ± SD (IQR)
Personal academic reputation	113 (3.1)	58 (1.6)	174 (4.8)	835 (22.9)	2460 (67.6)	4.50 ± 0.90 (1 - 5)
The reputation of the institution and academic community	64 (1.8)	76 (2.1)	268 (7.4)	1045 (28.7)	2187 (60.1)	4.43 ± 0.85 (1 - 5)
The reputation of the academic field	62 (1.7)	74 (2.0)	254 (7.0)	986 (27.1)	2264 (62.2)	4.46 ± 0.85 (1 - 5)
The normal progression of research activities	69 (1.9)	94 (2.6)	314 (8.6)	1045 (28.7)	2118 (58.2)	4.39 ± 0.89 (1 - 5)
The purity of scientific research	60 (1.6)	70 (1.9)	291 (8.0)	1033 (28.4)	2186 (60.1)	4.43 ± 0.85 (1 - 5)
The rational allocation of research resources	57 (1.6)	82 (2.3)	357 (9.8)	1110 (30.5)	2034 (55.9)	4.37 ± 0.87 (1 - 5)
The entire academic environment	54 (1.5)	77 (2.1)	345 (9.5)	1119 (30.7)	2045 (56.2)	4.38 ± 0.86 (1 - 5)
Public trust in scientists	47 (1.3)	62 (1.7)	293 (8.0)	998 (27.4)	2240 (61.5)	4.46 ± 0.82 (1 - 5)
Research integrity throughout the society	52 (1.4)	60 (1.6)	281 (7.7)	1035 (28.4)	2212 (60.8)	4.45 ± 0.82 (1 - 5)
Prestige in the individual's academic field	51 (1.4)	64 (1.8)	293 (8.0)	1024 (28.1)	2208 (60.7)	4.45 ± 0.83 (1 - 5)
Personal academic reputation	41 (1.1)	63 (1.7)	273 (7.5)	1040 (28.6)	2223 (61.1)	4.47 ± 0.80 (1 - 5)
Total score	-	-	-	-	-	48.80 ± 8.08 (11 - 55)

As shown in Table 3, the proportions of all seven optional reasons were high (six of the seven were above 60%). The top three perceived reasons were “nurses deviate in personal value and lack of academic ethics” (78.8%), “there is a lack of academic supervision” (72.6%), and “there exist defects of academic quantitative evaluation” (69.5%).

Overall, the percentages of “very strong influence” for all 11 listed perceived consequences were high (all were greater than 55%, Table 4). The average total score of perceived consequences for research misconduct was 48.80 ± 8.08.

## 2 Experimental Design, Materials and Methods

We applied the cross-sectional descriptive design. This study was approved by the Peking University Biomedical Ethics Committee (IRB00001052-18013). The inclusion criteria were as follows: (1) worked in a tertiary hospital; (2) had certification as a registered nurse; (3) informed consent to participate. Participants were excluded if they (1) could not participate in the study because of severe physical or mental diseases; (2) had more than three months of vacation in the past year; (3) worked in a position that was completely unrelated to nursing.

The questionnaire had five parts, including demographic characteristics, research activities, scientific misconduct knowledge, perceived reasons for research misconduct, and perceived consequences for research misconduct. As shown in Appendix I, demographic variables in the questionnaire included age, gender, marital status, years of career, fertility status, educational attainment, employment situation, title, institution level, department, and position.

The scientific misconduct knowledge questionnaire included six multiple-choice questions, which were designed according to a book published by the Research Integrity Construction Office of the Ministry of Science and Technology [6] and an official document released by the Ministry of Education of the People's Republic of China [7]. The knowledge questionnaire was designed mainly according to the chapter order of the book, which was similar to the common process of conducting research. Of the six questions, four were assessed perceived research misconduct during the process of writing research proposals, research project application, conducting research, and publishing papers, relatively; one was about authorship; one was about research ethics. We encoded each option (did not choose = “0”, choose = “1”) for the multiple-choice questions. The detailed scoring rules for each question are presented in the form of notes in Appendix I. For instance, the first question of the knowledge questionnaire has five options (A, B, C, D, and E), of which A and B are wrong answers. If you choose one of the wrong answers, then score “0”. Otherwise, one correct answer add one point. The total score of the knowledge questionnaire ranges from 0 to 30. Higher scores indicate higher knowledge levels.

A total of 11 yes-no questions were used to assess participants’ research activities, including publishing academic papers as the first author or corresponding author; publishing SCIE-indexed papers as the first author or corresponding author; publishing books as an editor-in-chief, etc. We also added up participation in 11 research activities as the research activity index to assess participants’ diversity and expertise of research.

The assessment tools of perceived reasons for research misconduct and perceived consequences for research misconduct have been applied in our previous study among graduate nursing students [8]. Perceived reasons were assessed by a multiple choice question with seven options. These designed perceived reasons for research misconduct options included “nurses are deviated in personal value and lack of academic ethics”, “nurses are lack of research ability”, “there is a lack of research integrity training”, etc. Perceived consequences were evaluated via an 11-item checklist, with a total score ranges from 11 to 55. Higher scores indicate more severe perceived consequences for research misconduct.

The data was downloaded from the online questionnaire system. We used SPSS 24.0 for data analysis. Descriptive analysis was conducted via mean and standard deviation (S.D.) for continuous variables, and frequency and percentages for categorized variables.

## Ethics Statements

Informed consent was collected from all the participants, and the research was carried out in accordance with the Declaration of Helsinki. This study was approved by the Peking University Biomedical Ethics Committee (IRB00001052-18013).

## CRediT Author Statement

**Shuyu Han:** Methodology, Roles/Writing – original draft, Formal analysis; **Ke Li:** Data curation, Software, Visualization, Writing – review & editing; **Zhiwen Wang:** Funding acquisition, Resources, Conceptualization, Supervision, Writing – review & editing. All the authors have approved the final version of submission.

## Declaration of Competing Interest

The authors declare that they have no known competing financial interests or personal relationships that could have appeared to influence the work reported in this paper.

## Data Availability

Dataset of research misconduct knowledge and associated factors among nurse in China: A national cross-sectional survey (Original data) (Mendeley Data).

## Appendix Supplementary materials

Image, application 1

Image, application 2
