# Supplementary material for: Dataset of research misconduct knowledge and associated factors among nurses in China: A national cross-sectional survey
Source: Data Brief. 2022 Jul 16;43:108471. doi: 10.1016/j.dib.2022.108471 (PMC9344333; doi:10.1016/j.dib.2022.108471)
Supplement: Supplementary file 1 [file mmc1.docx]

**Appendix I. Questionnaire**

**Part I. Demographic characteristics**

**Gender:**  A. Male B. Female

**Date of birth:** ____

**Marital status:** A. Unmarried B. Married

**Years of career:** ____

**Fertility status:** A. No B. Yes

**Educational attainment:** A. College or less B. Bachelor degree C. Master degree or above

**Employment situation:** A. Formal nurses B. Informal nurses

**Title:** A. Nurse B. Nurse practitioner C. Nurse-in-charge D. Associate director of nursing E. Director of nursing

**Institution level:** A. Grade A tertiary hospital B. Grade B tertiary hospital C. Grade C tertiary hospital

**Department:** A. Clinical department B. Nursing Department C. Other

**Position (Multiple choice):** A. Clinical position B. Research position C. Management position D. Service position

**Part II. Research activities**

Have you participated in the following scientific research activities after working? (Tick your answer for each item)

|  | **Yes** | **No** |
| --- | --- | --- |
| I have published academic papers as the first author or corresponding author |  |  |
| I have published SCIE-indexed papers as the first author or corresponding author |  |  |
| I have published books as a editor-in-chief |  |  |
| I have published books as editorial board |  |  |
| I am a PI for a research project |  |  |
| I have participated in research projects as other roles (non-PI) |  |  |
| I have won research awards |  |  |
| I have patents |  |  |
| I have attended academic conferences and have given oral or poster presentations |  |  |
| I am a reviewer for an academic journal |  |  |
| I am an editorial board member of an academic journal |  |  |

**Part III. Scientific misconduct knowledge questionnaire**

Which of the following is considered research misconduct when writing a project proposal? (Multiple choice)

1. The research topic is lack of innovation
2. The research method is unreasonable
3. Using other’s research program without permission
4. Exaggerating research protocols, implementation plans or experimental equipment
5. Violating ethical norms such as informed consent and privacy protection in research that involves human beings

*Note.* A and B are wrong answers. If choose one of them, then score “0”. Otherwise, one correct answer add one point.

Which of the following is considered research misconduct when applying for a research project? (Multiple choice)

1. Providing false information about the applicant's job title, academic qualifications, and research foundation
2. Forgery of application materials
3. Listing others as project members without notice and consent
4. Signing on behalf of other project members without consent

*Note.* All are correct answers. One correct answer add one point.

Which of the following is considered research misconduct during the research process? (Multiple choice)

1. Unintentional errors during the research process
2. Fabricating or falsifying raw data
3. Deleting raw data that out of expectations
4. Misunderstanding the research results
5. Untrue description of materials, instruments and equipment, and experimental processes in the research report
6. Using other's research methods, opinions, results or expressions without citation
7. Rewriting other's opinions or results without citation

*Note.* A and D are wrong answers. If choose one of them, then score “0”. Otherwise, one correct answer add one point.

Which of the following is considered research misconduct when publishing a paper? (Multiple choice)

1. Submitting papers to different journals at the same time
2. Repeating publication of the same study results in multiple papers
3. Signing other’s name without notice and consent

*Note.* All are correct answers. One correct answer add one point.

Which of the following can be involved into authorship when submitting a manuscript? (Multiple choice)

1. Superior as the corresponding author
2. Research plan designers
3. Research equipment providers
4. Research sample or specimen providers
5. People who provide general management for the research
6. Data Analysts
7. Funder of the research
8. Data collectors
9. Manuscript writers
10. Manuscript translators
11. People who provide language polishing

*Note.* C, D, E, G, J, and K are wrong answers. If choose one of them, then score “0”. Otherwise, one correct answer add one point.

Which of the following violate ethical principles in the human subjects studies? (Multiple choice)

1. Emphasizing or implying that the new method is superior to the control group
2. Emphasizing or implying that subjects will receive free medical treatment or fee subsidies if they accept the new method
3. Emphasizing or implying the academic authority of the experiment
4. Using inducing text, data, pictures, etc. to attract participants
5. Applying interventions without theoretical basis to the participants in the intervention group
6. Depriving intervention in the control group that deserved to have
7. Collecting research indicators in participants that may cause harm beyond normal treatment
8. Mentioning personal information of the participants to a person outside the research group
9. Mentioning information that can identify participants in the paper or in the photos of the manuscript
10. Information that identifies the participants appears on the slides during the academic presentations

*Note.* All are correct answers. One correct answer add one point.

**Part IV. Perceived reasons for research misconduct**

What is the reason do you think for academic misconduct? (Multiple choice)

1. Nurses are deviated in personal value and lack of academic ethics
2. Nurse are lack of research ability
3. There is a lack of research integrity training
4. Nursing do not understand the content of research integrity
5. There is a lack of academic supervision
6. There exist defects of academic quantitative evaluation
7. Nurse are influenced by social environment

**Part V. Perceived consequences for research misconduct**

To what extent do you think academic misconduct will affect? (Tick your answer for each item)

|  | **No influence** | **A little influence** | **Moderate influence** | **Strong influence** | **Very strong influence** |
| --- | --- | --- | --- | --- | --- |
| Personal academic reputation |  |  |  |  |  |
| The reputation of the institution and academic community |  |  |  |  |  |
| Reputation of the academic field |  |  |  |  |  |
| Normal progression of research activities |  |  |  |  |  |
| The purity of scientific research |  |  |  |  |  |
| Rational allocation of research resources |  |  |  |  |  |
| The entire academic environment |  |  |  |  |  |
| Public trust in scientists |  |  |  |  |  |
| Research integrity throughout the society |  |  |  |  |  |
| Prestige in the individual’s academic field |  |  |  |  |  |
| Other’s trust in for the researchers |  |  |  |  |  |
