# Supplementary material for: Dataset of research misconduct knowledge and associated factors among nurses in China: A national cross-sectional survey
Source: Data Brief. 2022 Jul 16;43:108471. doi: 10.1016/j.dib.2022.108471 (PMC9344333; doi:10.1016/j.dib.2022.108471)
Supplement: Supplementary file 2 [file mmc2.docx]

**Code book**

**Gender:** Male = “1”, Female = “2”

**Marital status:** Unmarried = “1”, Married = “2”

**Fertility status:** No = “1”, Yes = “2”

**Educational attainment:** College or less = “1”, Bachelor degree = “2”, Master degree or above = “3”

**Employment situation:** Formal nurses = “1”, Informal nurses = “2”

**Title:** Nurse = “1”, Nurse practitioner = “2”, Nurse-in-charge = “3”, Associate director of nursing = “4”, Director of nursing = “5”

**Institution level:** Grade A tertiary hospital = “1”, Grade B tertiary hospital = “2”, Grade C tertiary hospital = “3”

**Department:** Clinical department = “1”, Other = “2”

**Clinical position:** No = “0”, Yes = “1”

**Research position:** No = “0”, Yes = “1”

**Management position:** No = “0”, Yes = “1”

**Service position:** No = “0”, Yes = “1”

**RA1-RA11 (research activity):** No = “0”, Yes = “1”

**Knowledge1A - Knowledge1E, Knowledge2A - Knowledge2D, knowledge3A - Knowledge3G, Knowledge4A - Knowledge4C, Knowledge5A - Knowledge5K, knowledge6A - Knowledge6J:** Did not choose = “0”, Choose = “1”

**Reason1 - Reason7:** Did not choose = “0”, Choose = “1”

**Consequence1 - Consequence11:** No influence = “1”, A little influence = “2”, Moderate influence = “3”, Strong influence = “4”, Very strong influence = “5”
